# New Clothes for the Jasmonic Acid Receptor *COI1*: Delayed Abscission, Meristem Arrest and Apical Dominance

**DOI:** 10.1371/journal.pone.0060505

**Published:** 2013-04-01

**Authors:** Joonyup Kim, Bradley Dotson, Camila Rey, Joshua Lindsey, Anthony B. Bleecker, Brad M. Binder, Sara E. Patterson

**Affiliations:** 1 Department of Horticulture, University of Wisconsin, Madison, Wisconsin, United States of America; 2 Department of Biochemistry and Cellular and Molecular Biology, University of Tennessee, Knoxville, Tennessee, United States of America; 3 Department of Plant and Microbial Biology, University of California, Berkeley, United States of America; 4 Departamento de Ecosistemas y Medio Ambiente, Pontificia Universidad Católica de Chile, Santiago, Chile; 5 Orthopedics and Sports Medicine, University of Washington, Seattle, Washington, United States of America; 6 Department of Botany, University of Wisconsin, Madison, Wisconsin, United States of America; Instituto de Biología Molecular y Celular de Plantas, Spain

## Abstract

In a screen for delayed floral organ abscission in Arabidopsis, we have identified a novel mutant of *CORONATINE INSENSITIVE 1* (*COI1*), the F-box protein that has been shown to be the jasmonic acid (JA) co-receptor. While JA has been shown to have an important role in senescence, root development, pollen dehiscence and defense responses, there has been little focus on its critical role in floral organ abscission. Abscission, or the detachment of organs from the main body of a plant, is an essential process during plant development and a unique type of cell separation regulated by endogenous and exogenous signals. Previous studies have indicated that auxin and ethylene are major plant hormones regulating abscission; and here we show that regulation of floral organ abscission is also controlled by jasmonic acid in *Arabidopsis thaliana*. Our characterization of *coi1-1* and a novel allele (*coi1-37*) has also revealed an essential role in apical dominance and floral meristem arrest. In this study we provide genetic evidence indicating that *delayed abscission 4* (*dab4-1*) is allelic to *coi1-1* and that meristem arrest and apical dominance appear to be evolutionarily divergent functions for *COI1* that are governed in an ecotype-dependent manner. Further characterizations of ethylene and JA responses of *dab4-1*/*coi1-37* also provide new information suggesting separate pathways for ethylene and JA that control both floral organ abscission and hypocotyl growth in young seedlings. Our study opens the door revealing new roles for JA and its interaction with other hormones during plant development.

## Introduction

“*Falling peach blossoms*

*unfurling in the spring breeze*

*illuminating the way*…….”

from the Zen Master Eihei Dogen (1200–1253)

Just as the falling blossoms of the peach tree provided new insights to the Zen Master Dogen, it has been the prominent delayed abscission phenotype of *coronatine insensitive 1–37* (*coi1-37*) that is providing novel insights to basic plant development. Abscission, the detachment of organs from main body of a plant, is probably one of the earliest agricultural traits associated with crop domestication. In plants, abscission is a cell separation process where unwanted organs such as flower petals, sepals, and filaments are often shed after fertilization while pollen, seeds, fruits, and leaflets are shed in response to developmental cues or environmental conditions including pathogen attack or stresses [1], [2]. Both exogenous and endogenous signals regulate abscission. Plant hormones such as ethylene and auxin have long been recognized as endogenous factors regulating the timing of abscission [1], [3−8]. More recently, other endogenous signals important for abscission have been identified [9]–[17]. Although several molecules controlling abscission are now brought to light, there are many unanswered questions about how floral organ abscission is regulated. Therefore, we undertook a genetic approach to explore the factors that regulate floral organ abscission in Arabidopsis.

We previously screened over 32,000 Arabidopsis T-DNA insertion lines from the Wisconsin T-DNA collection [18] for delayed floral organ abscission and identified 17 *delayed abscission* (*dab*) mutants [14]. *dab4-1* was selected for further study because it retained its floral organs three to four times longer than wild type [3], [19]. Here, we further characterize *dab4-1* and show that it is allelic to *coi1-1* and that JA regulates the timing of floral organ abscission in Arabidopsis via an ethylene-independent pathway. Furthermore, we demonstrate that *DAB4/COI1* has additional roles in plant development including apical dominance and meristem arrest. These roles seem to represent evolutionarily diverged functions since they only appear in certain ecotype of Arabidopsis.

## Results

### Isolation and Phenotypic Analysis of Novel Delayed Floral Organ Mutants

**Figure 1:**
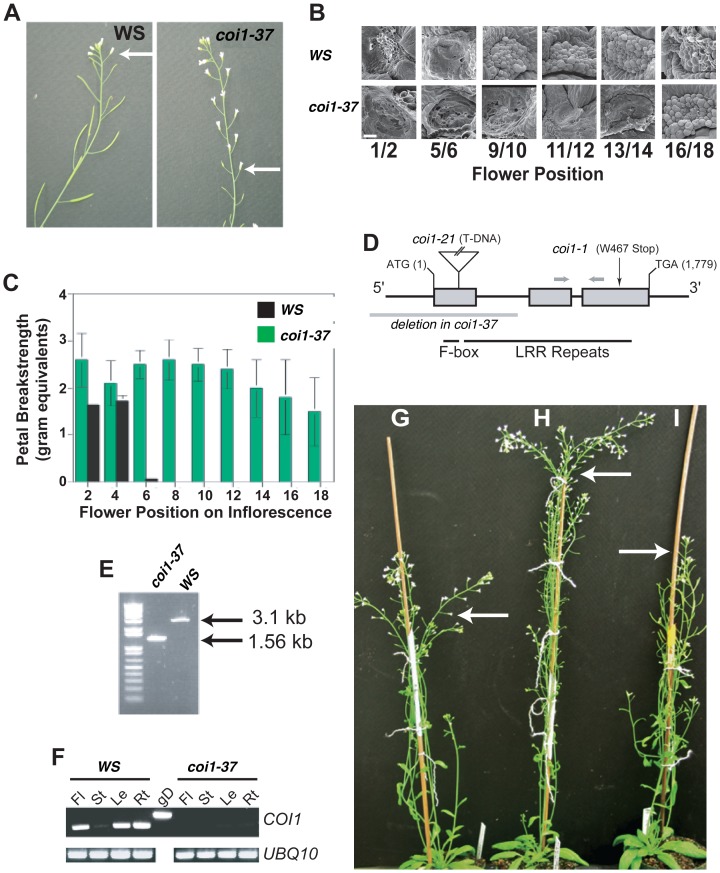
Isolation and Phenotypic, Molecular and Genetic Analysis of Novel *dab* Mutants. (A) Close-up of WS and *dab4-1* (*coi1-37)* inflorescences. Arrows indicate the flower position 7 in WS and 16 in *dab4-1* (*coi1-37)*. (B) Revealed fracture planes of the petal abscission zones were examined using scanning electron microscopy. Images were taken after petals were forcibly removed for each position. Flower positions as indicated are shown. Scale bar, 10 µm. (C) The force to remove a petal from each flower position was measured in *dab4-1* (*coi1-37)* and WS. (D) Diagram showing location of mutations for *coi1-37*, *coi1-21* and *coi1-1*. Primer locations for RT-PCR are denoted with arrows. (E) 1,537 bp deletion in *coi1-37* was examined using PCR and confirmed by sequencing the amplicons from both *coi1-37* and WS. (F) Expression of *DAB4*/*COI1* was examined in Fl (Flower), St (Stem), Lf (Leaf), and Rt (root) using RT-PCR. gD denotes genomic DNA amplicon using the same primer set. *UBQ10* was used as a loading control. (G-I) Genetic complementation of *coi1-37* with *coi1-21* and *coi1-1*. *coi1-21-*/- and *coi1-1*−/− were crossed to *dacoi1-37*+/−. Test cross analysis was performed to examine the delayed floral organ abscission phenotype was examined in the test cross population of (G) *coi1-21*−/− x *coi1-37*+/− and (H) *coi1-37*−/− x *coi1-17*+/− compared to WS (I).

Floral organ abscission typically progresses in a predetermined developmental sequence in Arabidopsis. In the current study, floral organs begin to abscise around flower positions 6–7 in the Wassilewskija (Ws) ecotype (Figure 1A). In contrast, the petals of *dab4-1* begin to abscise around position 17 (Figure 1A). Ultimately the floral organs abscised in *dab4-1*, but this delay was significantly longer than many other previously identified delayed abscission mutants including *etr1-1*, *ein2-1*, *dab1-1*, *2-1*, and *3-1* [14]. In addition, *dab4-1* has a similar degree of delay in the abscission process compared to other jasmonic acid and sterile mutants such as *aos* and *dde2-2 ein2-1* [19]. Scanning electron microscopy (SEM) of the revealed fracture plane shows that cellular changes such as rounding and elongation of the cells are normal in the *dab4-1* mutants, but that the timing of these changes is delayed (Figure 1B). Similarly, physiological tension measurements using a breakstrength meter for Arabidopsis [14], [20] reveal that the breakstrength pattern is prolonged in the *dab4-1* mutants and correlates with the position of abscission and SEM observations (Figure 1B, C). From these observations and measurements, we conclude that it is primarily the timing of abscission that is altered in *dab4-1* rather than a change or complete disruption in the abscission process.

By outcrossing to wild type, we determined that the delayed abscission phenotype of the *dab4-1* mutant is caused by a mutation in a single recessive locus (Table S1 and S2). All F1 plants were wild type and approximately one quarter of the segregating F2 plants had delayed abscission. In order to identify the genetic factor(s) for delayed floral organ abscission in the *dab4-1* mutant, we carried out map-based cloning. Two different F2 populations of *dab4-1* (WS) x Col and *dab4-1* (WS) x Ler were created for fine mapping. We determined that *dab4-1* has a 1,537 bp deletion in the promoter and the first exon comprising the F-box motif and the beginning of the leucine rich repeats of *COI1* (*CORONATINE INSENSITIVE 1*) (Figure 1D and 1E) [21]. Expression of *COI1* mRNA in *dab4-1* mutants was not detectible in the major tissues compared to wild type (Figure 1F) suggesting that there is no functional protein in the mutant.

To confirm the identity as *DAB4,* we examined *coi1-1* and isolated an additional allele from the SALK collection (Salk_035548, [22]). Homozygous lines of *coi1-1* and *SALK_035548 (coi1-21)* demonstrated delayed floral organ abscission comparable to *dab4-1.* In outcrosses the delayed abscission trait co-segregated with the genotype. We also performed test crosses of *coi1-1*−/− and *coi1-21*−/− to heterozygous *dab4-1*+/− plants, and the progeny segregated 1∶1 for wild type and delayed abscission indicating that *dab4-1* is allelic to *coi1-1* and *coi1-21* (Figure 1G-I). Additionally, *dab4-1* (hereafter *coi1-37*) was determined to be insensitive to meJA (Figure S1) as was previously observed in the original screen for *coi1-1* [23]. *coi1-37* also showed reduced mRNA levels for the genes involved in JA biosynthesis (Figure S2). Taken together, *DAB4*/*COI1* plays a key role in the timing of floral organ abscission.

### Novel Functions of *COI1*

**Figure 2:**
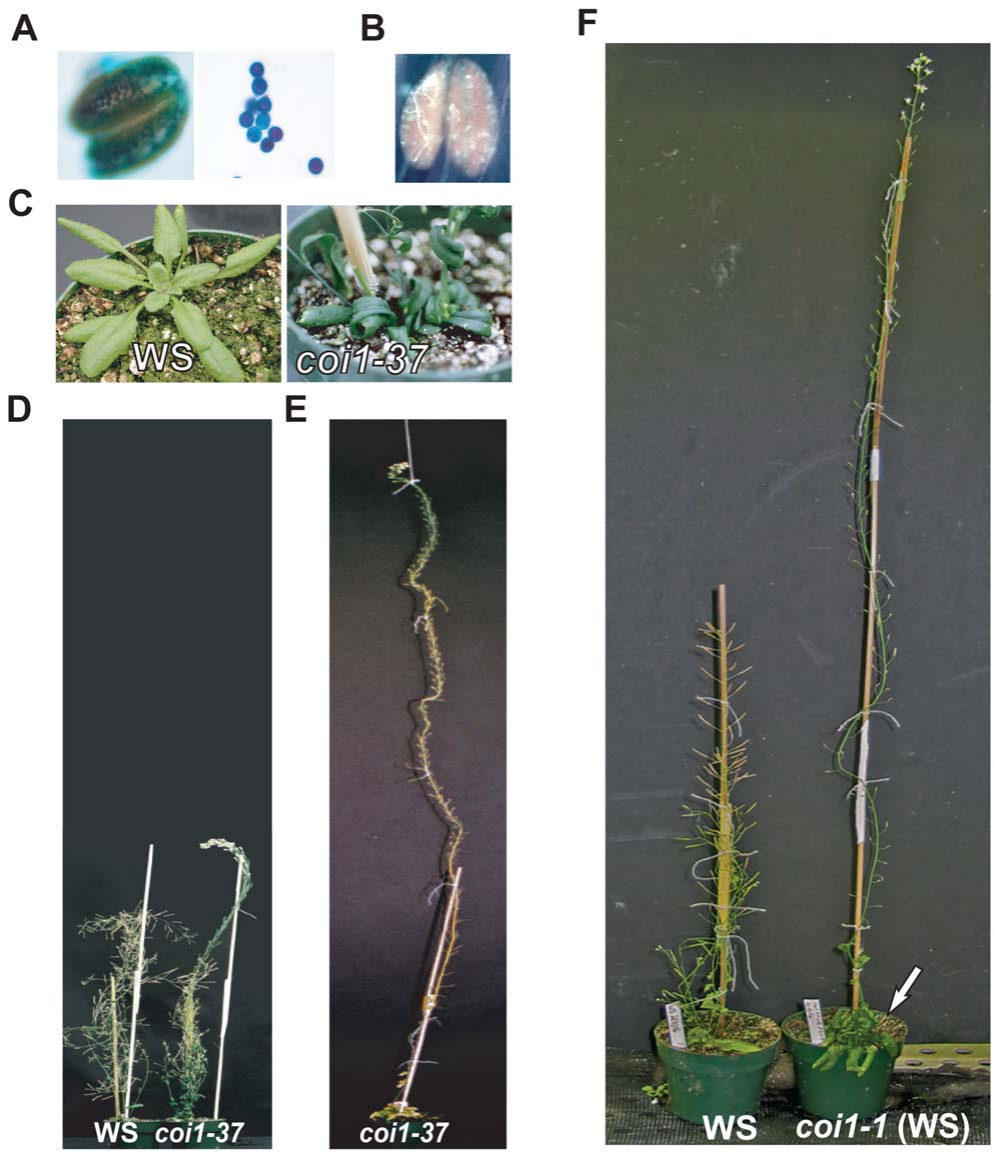
Additional Functions of *COI1* (*DAB4)* Governed by Specific Ecotype. (A) Pollen viability was examined in *coi1-37* (*dab4-1*). Pollen grains of *coi1-37* were stained with Alexander's stain in the anther (left) and removed from anther (right). (B) Germination of *coi1-37* pollen in the anther. Anthers were isolated from floral position 2 (after anthesis in wildtype) and examined with fluorescence microscopy. (C) Leaves of *coi1-37* compared to WS. Dark green and epinastic leaf growth are specific to WS ecotype of *dab4-1*. (D) Comparison of WS and *coi1-37* plants at 8 weeks after germination showing stronger apical dominance and continued proliferous growth in *coi1-37.* (E) *coi1-37* at 12 weeks after germination showing strong apical dominance and continued growth and flowering. (F) Comparison of 116d old WS to *coi1-1* outcrossed to WS. The *coi1-1* (WS) mutant acquired increased apical dominance and epinastic leaf growth. Arrow indicates epinastic and dark leaf growth in *coi1-1* (WS).

In addition to the regulation of timing of floral organ abscission, *coi1-37* mutants also display a range of developmental defects including male sterility, leaf epinasty, dark green leaves, strong apical dominance, and enhanced meristem longevity (Figures 2A–2F). Although approximately 50% of the pollen of *coi1-37 (dab4-1)* was viable (Figure 2A) and germinated on agar plates (data not shown), these plants were functionally male sterile due to a lack of dehiscence. We also observed that pollen germinated within the anther (Figure 2B). Particularly interesting was the strong apical dominance and delayed meristem arrest of *coi1-37* (Figure 2D-2F). In contrast to wild type plants that complete their life cycle within 12 weeks and produce approximately 35–55 flowers [24], *coi1-37* lives up to 4 months, growing almost two meters high and produces more than 300 flowers on the primary inflorescence (Figure 2E and 2F)

Interestingly, both *coi1-1* and *coi1-21* (both in Col ecotype) did not display other phenotypes such as strong apical dominance and epinastic leaf growth. In addition, we observed a reduction in the occurrence of strong apical dominance and epinastic leaf growth in the two mapping populations (*coi1-37* (WS) x Col and *coi1-37* (WS) x Ler). To better understand this, we outcrossed *coi1-1* (Col), which did not display the strong apical dominance or epinastic leaf growth, to WS and recovered these traits (Figure 2F) suggesting that the strong apical dominance and epinastic leaf growth of *coi1-37* is associated with *COI1* function and could be attributed to ecotype difference.

**Figure 3:**
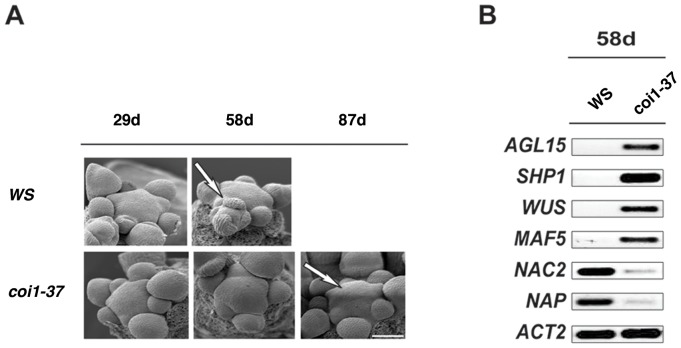
Inflorescence Meristem of *coi1-37* (*dab4-1*) is Indeterminate. (A) Inflorescence meristems were examined with SEM at different developmental stages after germination in WS and *coi1-37*. Arrow shows that WS inflorescence meristem is arresting while *coi1-37* meristem is still proliferating even at 87d after germination. SEM of wild type at “after-arrest” is missing since it already arrested completely. Scale bar, 10 µm. (B) Comparison of transcript levels of select genes from 58d old inflorescence meristem-enriched tissue from WS and *dab4-1* (*coi1-37*).

Our discovery of enhanced longevity with strong apical dominance in the *coi1-37* (WS) and *coi1-1* (WS) mutants prompted us to further examine the inflorescence meristem. The inflorescence meristems of *coi1-37* plants remained indeterminate and produced healthy flowers even after the wild type plant meristems started to become determinate and ultimately arrested. We examined the inflorescence meristems at 29d (before meristem arrest in WS), 58d (during meristem arrest in WS) and 87d (after meristem arrest in WS) (Figure 3A). SEM of the WS and *coi1-37* inflorescence meristems showed that there were no differences at 29d. However, morphological differences in the size and number of new floral bud primordia become quite apparent at 58d (Figure 3A). In addition, *coi1-37* continued to produce a turgid inflorescence meristem after 58 days while wild type plants stopped producing functional floral primordia (see also 87d in Figure 3A). This demonstrates that maintenance of inflorescence meristems in *coi1-37* is significantly prolonged; thus delaying the transition from indeterminate to determinate inflorescence.

In order to further understand the role of *COI1* in regulation of meristem integrity, we harvested inflorescence meristem-enriched tissue at 58d from wild type and *coi1-37* plants to perform a preliminary microarray analysis and confirmed in the following RT-PCR (Figure 3B and Figure S3). Previous studies have demonstrated that MADS box genes control major developmental stages in plants such as the development of gametophytes, embryos, seeds, roots, flowers and fruits [25]. Several of these MADS box genes determine floral organ identity and flowering time [25]–[28]. When constitutively expressed, *AGL15* (*AGAMOUS-like 15*) acts as a negative regulator of abscission and meristem longevity [29], *MADS AFFECTING FLOWERING 5* (*MAF5/AGL68*) acts as a potential floral repressor that is closely related to *FLOWERING LOCUS C* (*FLC*) [30], and *SHATTERPROOF 1* (*SHP1/AGL1*) acts as a negative regulator of dehiscence zone differentiation and floral organ abscission [31]. RT-PCR analysis consistently revealed that the expression levels for all three genes were up-regulated in *coi1-37* (Figure 3B and Figure S3).

Since *coi1-37* lives longer and can produce more than 350 flowers on the primary inflorescence, we also examined the expression of *WUSCHEL* (*WUS*). *WUS* controls stem cell fate throughout development and *WUS* loss-of-function mutants display a failure of self-maintenance in both shoot and inflorescence meristems [32], [33]. *WUS* expression was up-regulated in *coi1-37* inflorescence meristems relative to wild type (Figure 3B and Figure S3). In addition, the transcripts for two genes associated with senescence and apical/floral meristem regulation, *NAC-LIKE ACTIVATED BY AP3/PI* (*NAP*) and *NAC DOMAIN CONTAINING PROTEIN 2* (*NAC2*) [34]–[37], were significantly lower in *coi1-37* than wild type (Figure 3B and Figure S3). Regulation of these genes was similar when examined in JA biosynthesis and sterile mutant, *aos,* which displayed comparable floral organ abscission phenotype (Figure S2 and Figure S3) [19].

Together, these data suggest that JA signaling (or COI1) negatively regulates the levels of potential downstream target genes that are negative regulators of dehiscence and abscission as well as the expression of genes involved in meristem maintenance; and conversely, JA signaling (or COI1) enhances the levels of senescence-related genes. The mechanism for this COI1-mediated regulation of these downstream genes is unclear.

### Independent and Parallel Regulation of Floral Organ Abscission by JA and Ethylene

COI1 has recently been identified as a JA co-receptor in Arabidopsis [38], [39]. Molecular and genetic studies on *coi1* mutants suggest that COI1 is involved in jasmonate-signaled defense responses and other aspects of plant growth including pollen dehiscence, maternal control of seed maturation, and glandular trichome development [40], [41]. Our observations on *coi1-37* reveal that JA has more encompassing roles in plant development than traditionally recognized. In re-evaluating published reports we found that JA biosynthesis mutants such as *fad3-2 fad7-2 fad8* triple mutant, *dad1 (defective in anther dehiscence 1)*, *dde1(delayed dehiscence 1)*, and *aos* (*dde2*) (*allene oxidase synthase)* displayed comparable delayed abscission similar to *coi1-37, coi1-21 and coi1-1* [19], [42]–[45].

**Figure 4:**
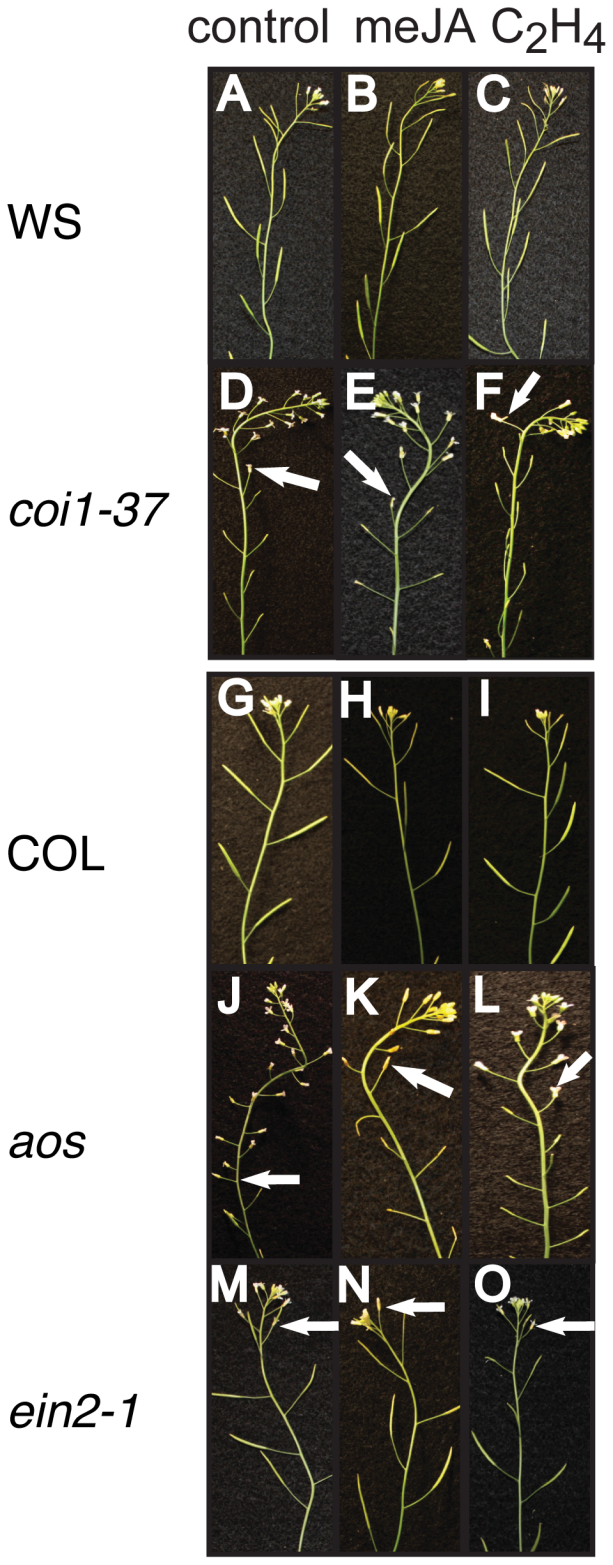
JA Signaling and Biosynthesis Regulate Floral Organ Abscission. 42d-old plants were treated with either 200 µM meJA or 1 ppm ethylene. Air treated plants were used as controls. Arrows indicate the last floral positions with petals still attached. (A–C) Responses of WS to (A) air, (B) meJA and (C) ethylene. (D–F) Responses of *coi1-37* to (D) air, (E) meJA and (F) ethylene. *coi1-37* shows accelerated floral organ abscission only with ethylene treatment as indicated with arrows. (G–I) Responses of Col to (G) air, (H) meJA and (I) ethylene. (J–L) Responses of *aos* to (J) air, (K) meJA and (L) ethylene. Both applications accelerated floral organ abscission. (M–O) Responses of *ein2-1* to (M) air, (N) meJA and (O) ethylene. *ein2-1* shows accelerated floral organ abscission only to meJA, but unresponsive to ethylene.

To better understand the role of JA during floral organ abscission, we applied 200 µM meJA to flowering Arabidopsis. Application of meJA accelerated the timing of floral organ abscission (by 2–3 flower positions) in wild type, but not in *coi1-37* (Figure 4A-B and 4D-E). However, acceleration of floral organ abscission by 5–6 flower positions was observed with the *aos* (*allene oxide synthase*) JA biosynthesis mutant when 200 µM meJA was exogenously applied (Figure 4G-H and 4J-K). Since ethylene is known to accelerate the timing of abscission and ethylene insensitivity is associated with delayed abscission [14], we examined the effects of ethylene on floral organ abscission in these same mutants. Interestingly, application of ethylene accelerated floral organ abscission of both *coi1-37* and *aos* (Figure 4C, 4F, 4I and 4L). Consistent with these results, both meJA and ethylene accelerated leaf senescence in WS and COL while only ethylene was effective at accelerating senescence of *coi1-37* leaves (S4). These results indicate that normal JA biosynthesis or signaling is not required for ethylene's effect on abscission since ethylene accelerates floral organ abscission of both mutants (Figure 4, Figure S2 and S4). In contrast, the *ein2-1* ethylene insensitive mutant [14] had accelerated abscission when treated with meJA (by 2–3 flower positions) but not when treated with ethylene (Figure 4M-O and Figure S5). In conclusion, the perception of ethylene is not required for JA dependent floral organ abscission, and we conclude that ethylene and JA accelerate floral organ abscission in Arabidopsis via independent pathways. This is consistent with the roles for these two hormones in the growth responses in seedlings [46].

### Cross Talk between Ethylene and JA in Hypocotyl Growth

**Figure 5:**
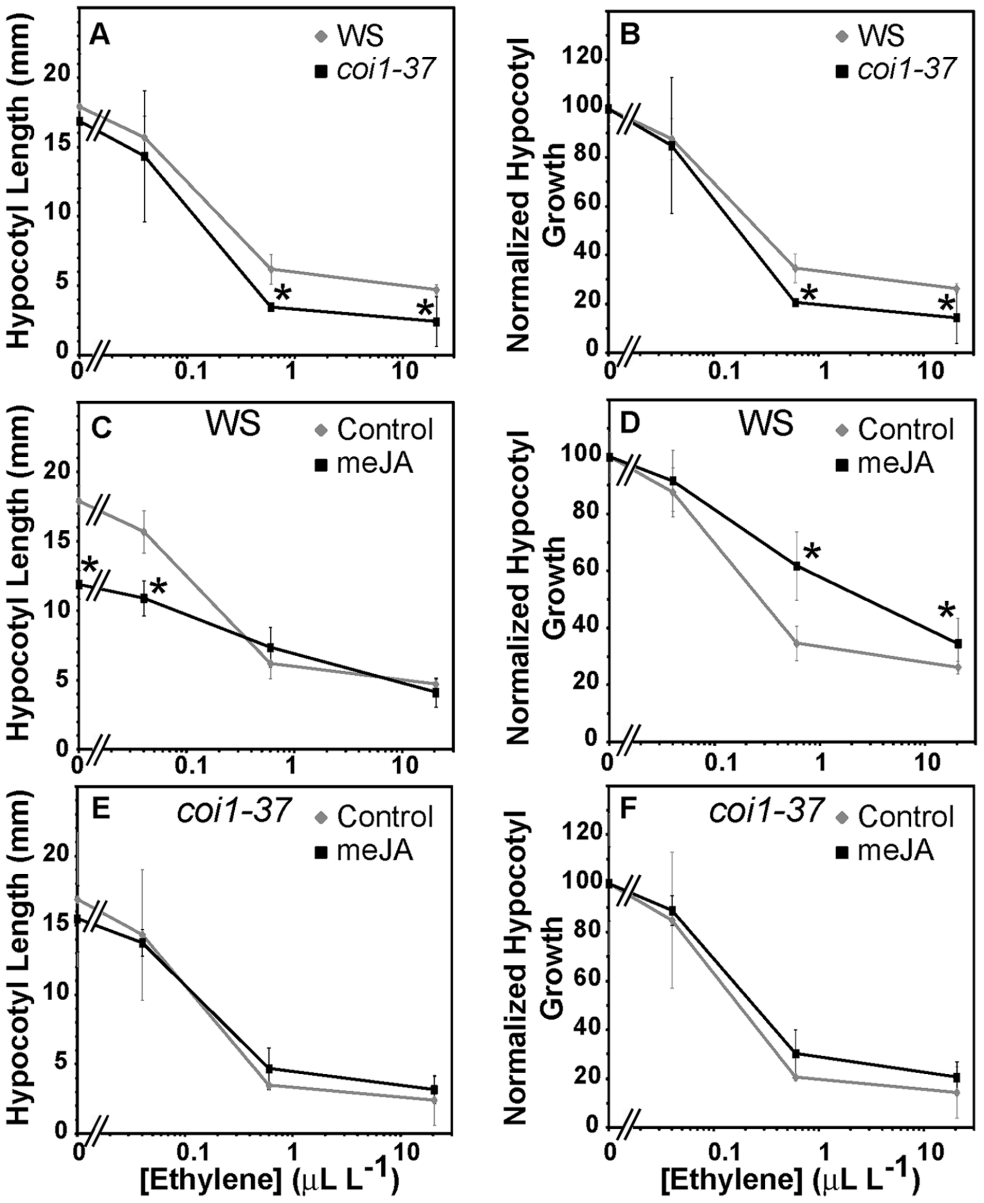
Dose-responses of Dark Grown Seedlings to Ethylene and JA. Dark-grown seedlings were treated with the indicated concentration of ethylene for 3 days and the length of the hypocotyls were measured to determine the ethylene-dose responses (A,C,E). Data was also normalized (B,D,F) to determine the relative responsiveness to ethylene at various dosages. The error bars represent standard deviation. Responses at a particular concentration of ethylene were compared using a t test and considered statistically significant if *P*<0.05. (A-B) Ethylene dose-responses of WS and *coi1-37* seedlings in the absence of applied meJA. * indicates significant difference from WS (*P* <0.05). (C-D) The ethylene dose-responses of WS in the absence and presence of 10 µM meJA. * indicates significant difference from untreated control (*P* <0.05). (E-F) The ethylene dose-responses of *coi1−37* in the absence and presence of 10 µM meJA.

To extend our understanding about the biological functions of COI1, we examined the responses of *coi1-37* mutants to various plant hormones in other aspects of plant development. Wild type and *coi1-37* seedlings showed no obvious differences in responses to auxin, brassinolides, cytokinins, or gibberellins (data not shown). As described above, we found that both the *coi1-37* and *aos* mutants were responsive to exogenous ethylene. We further examined possible interactions between JA and ethylene by examining the effects of JA on the growth inhibition of dark-grown seedlings caused by different dosages of ethylene (Figure 5). This assay has previously been useful in quantifying responses to ethylene in wild type and mutant seedlings [47], [48].

Consistent with prior reports [49], [50], we found that ethylene inhibited hypocotyl growth of wild type seedlings with a half-maximal dosage of approximately 0.2 ppm (Figure 5A,B). In air, the length of the *coi1-37* hypocotyls was indistinguishable from WS seedlings (Figure 5A). Unexpectedly, *coi1-37* mutants were somewhat more responsive to ethylene than WS hypocotyls at higher ethylene concentrations (Figure 5A, B). We also examined the effects of 10 µM meJA on ethylene responses (Figure 5C-F). In wild type seedlings, application of meJA reduced the length of hypocotyls approximately 33% in air and at low ethylene concentrations (Figure 5C). However, at higher ethylene concentrations there was no apparent effect of meJA. When these data were normalized, we observed that meJA reduced responses to ethylene at higher ethylene concentrations (Figure 5D). This is interesting since it is opposite to what was observed in the JA insensitive *coi1-37* mutants (Figure 5B). In contrast to wild type seedlings, meJA had no significant effect (*P* <0.05) on the ethylene dosage-response of *coi1-37* hypocotyls (Figure 5E and F). In summary, JA signaling appears to modulate seedling responses to ethylene in regulation of hypocotyl growth.

## Discussion

The role of ethylene during abscission was determined as early as the 1900s when Neljubov observed that it was the ethylene released from gas streetlights that caused early leaf abscission on nearby trees [51]. In general, scientists recognize that ethylene promotes abscission, while auxin delays this process [1]–[8]; however the role of jasmonic acid during this process is often overlooked. Recent identification and characterization of novel mutants in rice and Arabidopsis has also begun to shed light on other endogenous signal molecules important for abscission [4], [9]–[13], [15]–[17], [52], [53]. While a role in dehiscence was initially identified for *COI1*, the essential role in the important biological and agricultural process of abscission was not highlighted [23], [38]–[40]. In addition, earlier studies on the efficacy of jasmonates in regulating abscission in horticultural crops has been mixed [54]. However, several researchers have reported that application of meJA hastened abscission (or loosening of fruit) in crops such as citrus and tomato [54]–[58]. Since many of these studies addressed the role of JA in combination with other compounds including silver nitrate, ethylene and 1-MCP, this may explain why the role of JA in abscission has not been studied more extensively. Even in recent prominent review articles, this important role of jasmonic acid during abscission is not emphasized [59], [60].

**Figure 6:**
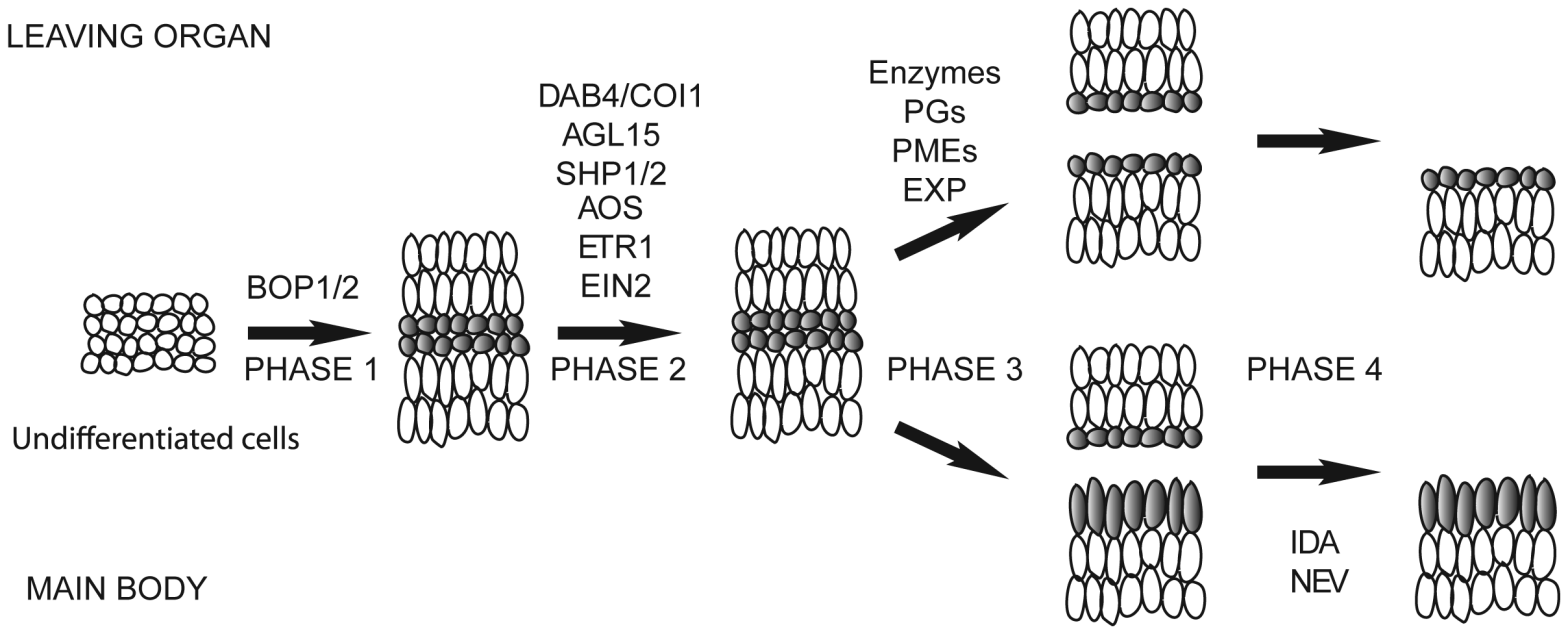
Proposed Model of Regulation of Floral Organ Abscission, Inflorescence Meristem Maintenance, and Senescence by *COI1*. Working model of floral organ abscission. Based on the progression, in one model, floral organ abscission can be divided into 4 phases. Phase 1, pre-abscission phase where abscission zones (organ boundaries) are established in the early development, Phase 2 where abscission cells acquire competence to abscission signals while structurally dissolution of middle lamellae is observed, Phase 3 where abscission cells are activated when cell wall loosening is occurring, and Phase 4 where cell repair is observed in the post abscission trans-differentiation phase. Previously studied regulators in floral organ abscission are included in this model: *DAB4/COI1*. *BOP1/2* (*BLADE ON PETIOLES 1/2*), HWS (HAWAIIAN SKIRT), *PGs* (*Polygalacturonases*). PMEs (*Pectin* methyltransferases), *EXP* (*Expansins*), *IDA* (*INFLORESCENCE DEFICIENT IN ABSCISSION*) and *NEV* (*NEVERSHED*).

In this study, we isolated and characterized the novel *delayed abscission* mutant *dab4-1/coi1-37* as well as *coi1-1* and showed that *DAB4/COI1* is required for proper floral organ abscission. The *coi1-37* mutant shows a significant delay in floral organ abscission; yet still progresses to ultimately abscise organs. In *coi1-37* plants, the abscission process is delayed, yet has basic cellular morphology similar to wild type as evidenced by both longitudinal sections and SEMs. We previously proposed a model (Figure 6) for abscission that consists of four major steps: pre-determination of abscission cells before differentiation (Phase 1), acquisition of competence to respond to signals such as ethylene (Phase 2), activation of process (Phase 3), and post abscission trans-differentiation (Phase 4) [2]. Based on this current study, we propose that that JA signaling participates in Phase 2 to regulate the proper timing of abscission (Figure 6). In this model, we propose that ethylene and JA are acting in parallel pathways to accelerate abscission.

Our characterization of *coi1-37*, *aos* and *ein2-1* in response to JA and ethylene provides evidence of crosstalk between JA and ethylene during the abscission process. Similarly, our results using the growth inhibition of effects of ethylene in seedling assays also suggest an interaction between ethylene and JA. *coi1-37* mutants were more responsive to ethylene at higher ethylene dosages while application of meJA desensitized seedlings to higher dosages of ethylene. This provides insights into JA and ethylene interactions as *coi1* seedlings are insensitive to meJA. There have been studies on cross talk between JA and ethylene that have suggested that JA and ethylene act both synergistically and antagonistically in the regulation of plant defenses [61], [62]. Our study also supports the idea that JA desensitizes the plant's ability to sense further stress signals such as ethylene in regulation of hypocotyl elongation affecting downstream signal cascade. This altered responsiveness to ethylene in the presence of meJA required JA perception as the *coi1-37* mutants showed no significant alterations in seedling ethylene responses. This is consistent with prior experiments showing that meJA-mediated growth reduction in root and alterations in the curvature of the apical hook of dark grown seedlings was *COI1*-dependent [63]. The exact mechanism for this crosstalk between JA and ethylene is not clear but may involve *ERF1* expression (Figure S3C) or expression of the EIN3/EIL1 transcription factors [64]–[66].

Particularly interesting was the observation that *coi1-37* mutants have leaf epinasty, dark green leaves and stems, strong apical dominance, and enhanced meristem longevity. Two traits, delayed abscission and male sterility (due to a lack of anther dehiscence), were observed in *coi1-37* mutants in the Col, Ler, and WS backgrounds. However, the other traits were ecotype-dependent and were only observed in *coi1* mutants in the WS background. Inflorescence meristem arrest is tightly associated with both plant life cycle and the plant's ability to produce and eventually abscise flowers. Generally wild type Arabidopsis ceases floral initiation on the primary inflorescence after production of 35–45 flowers; and while new floral organs are reinitiated if all the developing siliques are removed, arrest occurs within several days after producing an additional 10–12 flowers [24]. While *coi1-37* is infertile, this is not likely to be the major factor regulating its longevity. For example, the primary inflorescence of another infertile mutant, *male sterile1*, terminates after producing 55–75 flowers [24]. In addition, fertilizing *dab4-1* flowers did not significantly alter either the timing of abscission or longevity (data not shown).

One explanation for these observations could be that floral organ abscission and anther dehiscence are more ancient traits associated with *DAB4/COI1* and are conserved in ecotypes such as WS, Col, and Ler. In contrast, strong apical dominance and epinastic leaf growth may represent more recently diverged functions for *DAB4/COI1* that were either lost in Col and Ler or gained in WS. Possible genetic modifiers that provide additional functions for *DAB4* still remain to be identified. Our gene expression results indicate that the enhanced longevity of *dab4-1* plants is also associated with an up-regulation of specific MADS box genes and WUS as well as down-regulation of senescence-related and apical meristem regulation genes. Further investigations will provide novel insights on the relationship of JA with meristem associated genes such as the MADS box genes in the regulation of floral organ abscission and plant longevity.

## Experimental Procedures

### Plant Materials and Growth

*Arabidopsis thaliana* of Columbia (Col), Wassilewskija (WS) and Lansberg erecta (Ler) ecotypes were used for this study. The *dab4-1* (*coi1-37*) mutant was isolated from the Wisconsin T-DNA lines and additional T-DNA insertion mutants were isolated from SALK resources (http://signal.salk.edu/cgi-bin/tdnaexpress). Plants were grown as described by Patterson and Bleecker [14].

#### Map-based Cloning

*dab4-1* in the Wassilewskija (WS) ecotype was outcrossed to Columbia (Col) and Lansberg erecta (Ler) ecotypes to create the initial mapping populations. *dab4-1* in the F2 population from each cross was utilized for further backcrosses to WT. Fine mapping was conducted with *dab4-1* (WS) X Col and *dab4-1* (WS) X Ler F2 population. DNAs from more than 1,400 individual *dab4-1* plants were collected and used to localize *DAB4 (COI1)*.

#### Scanning Electron Microscopy

Samples were harvested and immediately fixed in 4% gluteraldehyde in a 0.05M KPO4 buffer pH7.2. After 4–12 hours fixation at 4°C, samples were dehydrated with an ethanol series and critical dried in liquid carbon dioxide at the SEM facility (Animal Science UW, Madison). Samples were sputter coated with gold palladium and viewed on a (Hitachi S-570; Hitachi Ltd.,Tokyo, Japan) at an accelerating voltage of 10 kV.

#### Petal breakstrength analysis

Petal breakstrength measurements were taken on flowers from the primary inflorescences of *dab4-1 (coi1-37)* and WS plants approximately one week prior to growth arrest. Measurements were taken beginning at flower position 1 (anthesis) and for each subsequent flower. Breakstrength measurements were conducted on a stress transducer developed by Edgar Spalding and Anthony Bleecker (Botany Department, University of Wisconsin, Madison). A small alligator clamp was attached to petals at each position, and the minimum force required to remove each individual petal was measured by using a modified FORT 10 force transducer (World Precision Instruments, Inc., Sarasota, FL.) and a voltmeter (Radio Shack, Fort Worth, TX). Readings were taken in millivolts and converted to gram equivalents before graphing.

#### Pollen viability test

In order to verify pollen viability, pollen was stained with Alexander's stain (Alexander 1969). This staining solution was prepared by mixing 10 ml ethanol, 1 ml 1% malachite green dissolved in ethanol, 50 ml distilled water, 25 ml glycerol, 5 g phenol, 5 ml 1% acid fuchsin dissolved in water, 0.5 ml 1% orange G dissolved in water, and 3 ml glacial acetic acid. Aborted pollen appears green, while viable pollen stains red to deep red depending upon the material, concentration of the dyes and pH of the medium. All samples were immediately observed on an Olympus BX60 microscope (Olympus Optical Co., Tokyo, Japan).

### RNA Isolation and RT-PCR

Total RNA was extracted using a TRIzol Reagent (Invitrogen, Carlsbad, CA) from at least three biological samples of inflorescence apex enriched tissues, stems, and leaves of WS and *dab4-1*. The quality and quantity of the extracted RNA were examined on a 1.2% (w/v) Agarose gel and by using a Nanodrop (ND-1000) spectrophotometer (Thermo Fisher Scientific, Pittsburgh, PA). One µg of total RNA was used for cDNA synthesis using ImProm-II™ Reverse Transcription System (Promega, Madison, WI) according to the manufacturer's instructions. The RNA was treated with one unit of DNase I (Invitrogen, Carlsbad, CA) prior to cDNA synthesis, and the cDNAs were diluted 10-fold. Resulting cDNAs were diluted 1∶10 and 2 µl was added as template for a standard 20 µl PCR reaction. All reactions were incubated at 95°C for 3 minutes, and cycled 28 or 36 times as follows: 95°C for 30 seconds, annealing temperature (58–62 °C) for 45 seconds, 68°C for 1 minutes. After the last cycle, reactions were incubated at 68°C for 5 minutes. qRT-PCR was performed as previously described [67]. Primer sequences used for RT-PCR are available in Table S3.

#### DNA Sequencing

In order to identify the deleted region of *dab4-1(coi1-37)* in *COI1*, PCR products amplified using primers Forward- GTTCTTTGTAAGTGTGGTCCGAGT and Reverse- AGCAAGCATAACAGTTGCAAAGC were excised from the gel, and purified using a Quiagen Gel extraction kit (Qiagen,Valencia, CA). Purified products were sequenced directly using cycle sequencing and fluorescently labeled dideoxy terminators (BIG DYE Applied Biosystems, Foster, CA). All reactions were outsourced to the UW-Madison Biotechnology Center and run on an ABI automated DNA sequencer.

#### JA and Ethylene Treatments

To determine JA effects on roots, seedlings were grown in the light on half strength MSNS plates for 5 days with or without 10 µM meJA and root length measured. Ethylene responses were observed on seedlings grown in the dark for 3d in sealed containers with constant flow at 100 ml min^−1^ of either hydrocarbon-free air or the indicated concentration of ethylene. Application was performed in dark closed chambers. Alternatively for whole plant assays with ethylene and air, flowers, leaves, and inflorescence apex tissues were grown in sealed containers with constant flow at 100 ml min^−1^ of either hydrocarbon-free air or 1 ppm ethylene for 3d. For whole plant JA assays, plants were grown in similar containers and sprayed twice a day for three days with 200 µM meJA. Plants were observed for leaf senescence, petal abscission, and meristem arrest.

## Supporting Information

Figure S1 **MeJA affects WS but not** ***coi1-37*** **seedlings.** Seedlings were grown on ½ MSNS plates under long day conditions (16 h light, 8 h dark) for 5 days in the presence or absence of 10 µM of meJA as shown. Scale bar, 5 mm.
(TIF)

Figure S2 **Transcript levels of JA biosynthesis genes in** ***coi1-37*** **.** Stems and leaves from wild type (WS) and *coi1-37* were harvested for RT-PCR analysis. Tissues were collected at the same age (58 days) as inflorescence meristem analysis shown in Figure 3. Three major JA biosynthesis genes (*DDE1*, *AOS*, and *DAD1*) were examined for the transcript levels. Among others, *DAD1* gene was not detectible for its transcript.
(TIF)

Figure S3 **Transcript abundance in JA mutants.** Inflorescence meristem-enriched tissues from wild type (Ws and Col), *coi1-37*, and *aos* were harvested for qRT-PCR analysis. Tissues were collected at the same age (58 days) as shown in Figure 3. Transcript levels of potential downstream targets (*AGL15*, *SHP1*, *WUS*, *MAF5*, *NAC2*, and *NAP*) (A, B, D, E) and ethylene signaling genes (*ERF1* and *ERS1*) (C) relative to *ACT2* were normalized to corresponding wild type used. Transcript levels of potential downstream targets in JA signaling mutant, *coi1-37* in Ws background (A and B) and JA biosynthesis mutant, *aos* (D and E) were analyzed. Data represent the average ± SEM from three biological replicates.
(TIF)

Figure S4 **Leaf senescence of JA mutants and ethylene mutant** ***ein2-1*** **.**Comparison of leaves from wild type WS and Col to *coi1-37*, *aos* and *ein2-1*. Leaves were treated with 200 µM of meJA and 1 ppm ethylene as designated in Experimental Procedures. While *aos* displayed senescent tissues in response to both application of meJA and ethylene, *coi1-37* and *ein2-1* were only responsive to ethylene and meJA respectively. Wild type WS and Col displayed senescence with application of both meJA and ethylene.
(TIF)

Figure S5 **JA-dependent floral organ abscission.** Comparison of floral organ abscission in Col (A-C) and *ein2-1* (D-F). Whole plants are applied with 200 µM of meJA and 1 ppm of ethylene as described in Materials and Methods. Images are taken from primary inflorescence from Col and *ein2-1*. While Col was responsive to the applications of both meJA and ethylene (note the decreased numbers of flowers with petals still remain attached as well as total flowers), *ein2-1* was only responsive to meJA (E). Both treatments accelerated floral organ abscission by position by 2-3 in wild type (B and C). The comparable degree of acceleration of abscission was observed in *ein2-1* with responses to meJA (by flower position 3). Arrows indicate flower positions 4 (E) and 7 (D and F) of *ein2-1* inflorescence respectively.
(TIF)

Table S1 Segregation of delayed floral organ abscission trait in F2 population of *coi1-37*.
(DOCX)

Table S2 Segregation of delayed floral organ abscission trait and apical dominance traits in F1 population of test crosses.
(DOCX)

Table S3 Primers used for semi-quantitative and quantitative RT-PCR analysis in *coi1-37*.
(DOCX)
