# Correction: New Clothes for the Jasmonic Acid Receptor *COI1*: Delayed Abscission, Meristem Arrest and Apical Dominance

**DOI:** 10.1371/journal.pone.0119063

**Published:** 2015-03-25

**Authors:** The PLOS ONE Staff

S4 Fig incorrectly appears as a duplicate of File S5. Please view the correct file, which has been renamed S4 Fig below.

## Supporting Information

S4 Fig Leaf senescence of JA mutants and ethylene mutant *ein2–1*. Comparison of leaves from wild type WS and Col to *coi1–37*, *aos* and *ein2–1*. Leaves were treated with 200 μM of meJA and 1 ppm ethylene as designated in Experimental Procedures. While *aos*displayed senescent tissues in response to both application of meJA and ethylene, *coi1–37* and *ein2–1* were only responsive to ethylene and meJA respectively. Wild type WS and Col displayed senescence with application of both meJA and ethylene.
(TIF)
