# Supplementary figures and images for: New Clothes for the Jasmonic Acid Receptor COI1: Delayed Abscission, Meristem Arrest and Apical Dominance
Source: PLoS One. 2013 Apr 1;8(4):e60505. doi: 10.1371/journal.pone.0060505 (PMC3613422; doi:10.1371/journal.pone.0060505)

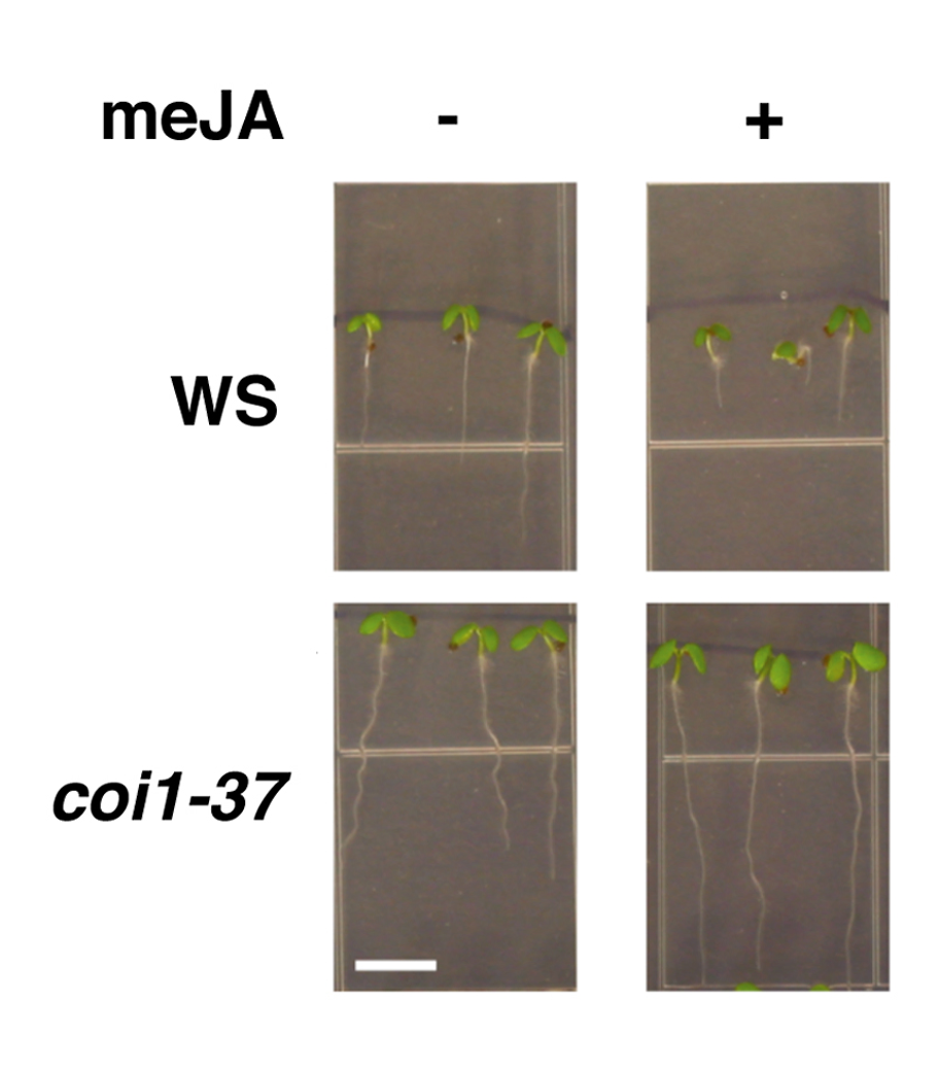

Supplement: Figure S1 — MeJA affects WS but not coi1-37 seedlings. Seedlings were grown on ½ MSNS plates under long day conditions (16 h light, 8 h dark) for 5 days in the presence or absence of 10 µM of meJA as shown. Scale bar, 5 mm. (TIF) [file pone.0060505.s001.tif]

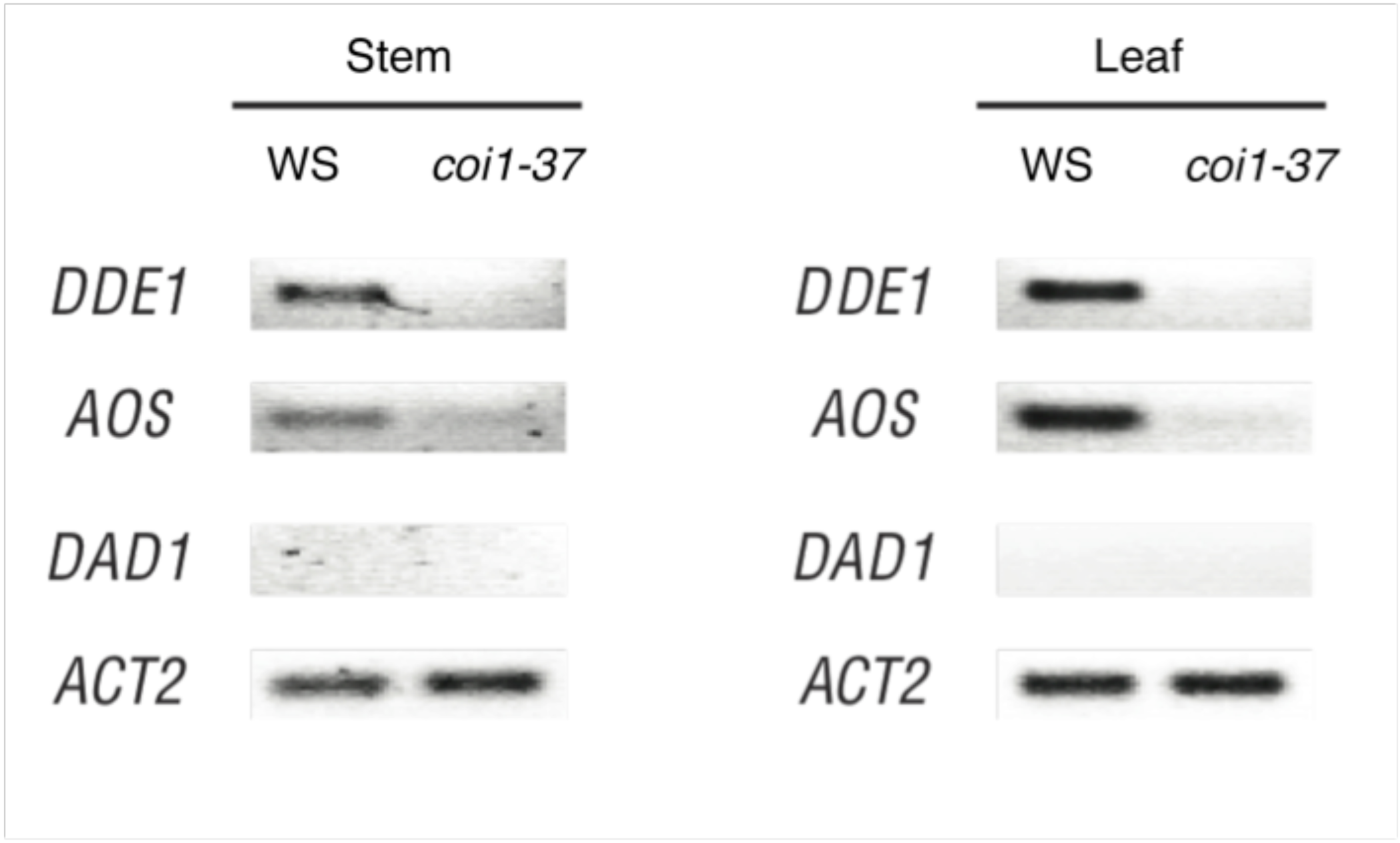

Supplement: Figure S2 — Transcript levels of JA biosynthesis genes in coi1-37 . Stems and leaves from wild type (WS) and coi1-37 were harvested for RT-PCR analysis. Tissues were collected at the same age (58 days) as inflorescence meristem analysis shown in Figure 3. Three major JA biosynthesis genes (DDE1, AOS, and DAD1) were examined for the transcript levels. Among others, DAD1 gene was not detectible for its transcript. (TIF) [file pone.0060505.s002.tif]

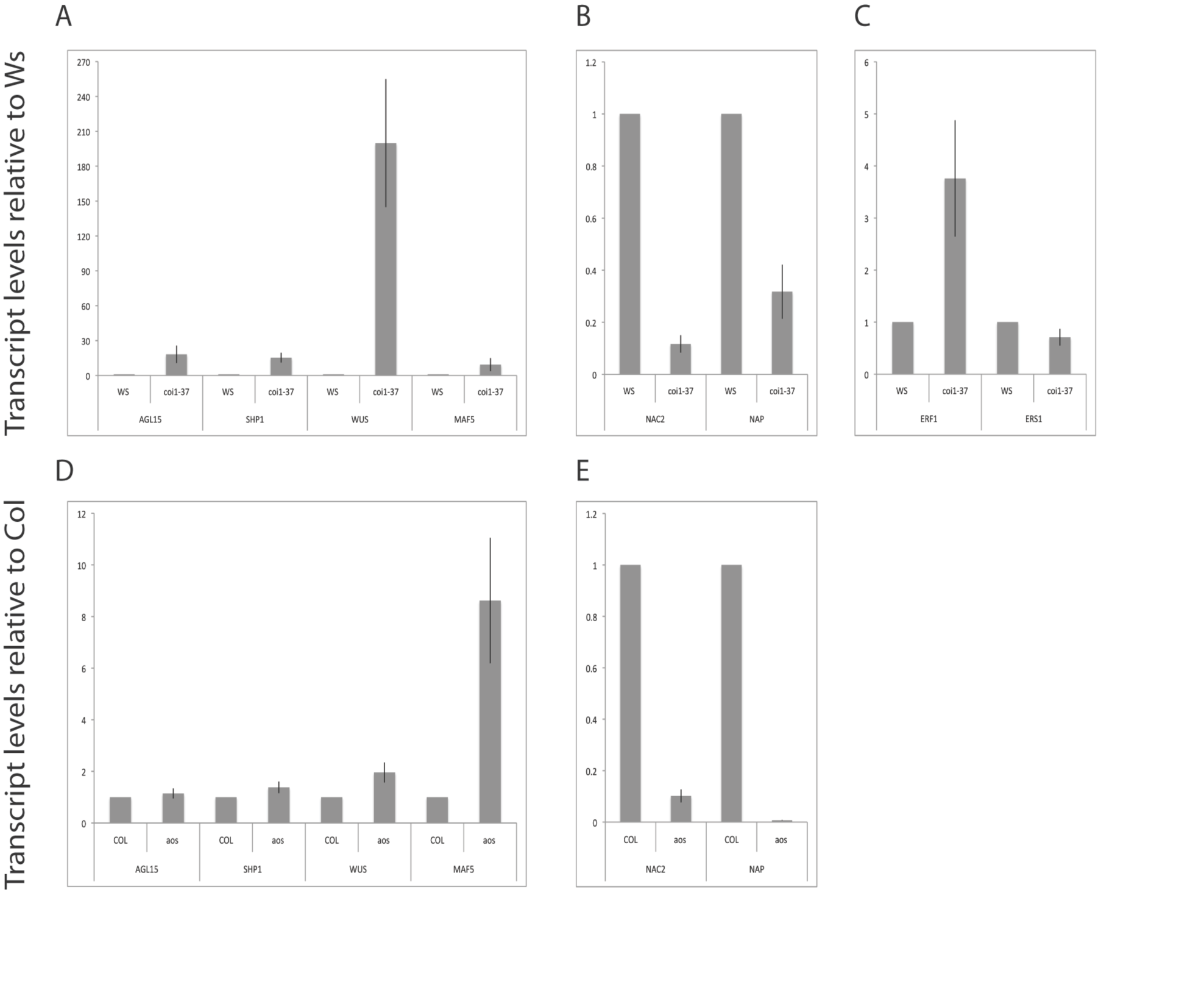

Supplement: Figure S3 — Transcript abundance in JA mutants. Inflorescence meristem-enriched tissues from wild type (Ws and Col), coi1-37, and aos were harvested for qRT-PCR analysis. Tissues were collected at the same age (58 days) as shown in Figure 3. Transcript levels of potential downstream targets (AGL15, SHP1, WUS, MAF5, NAC2, and NAP) (A, B, D, E) and ethylene signaling genes (ERF1 and ERS1) (C) relative to ACT2 were normalized to corresponding wild type used. Transcript levels of potential downstream targets in JA signaling mutant, coi1-37 in Ws background (A and B) and JA biosynthesis mutant, aos (D and E) were analyzed. Data represent the average ± SEM from three biological replicates. (TIF) [file pone.0060505.s003.tif]

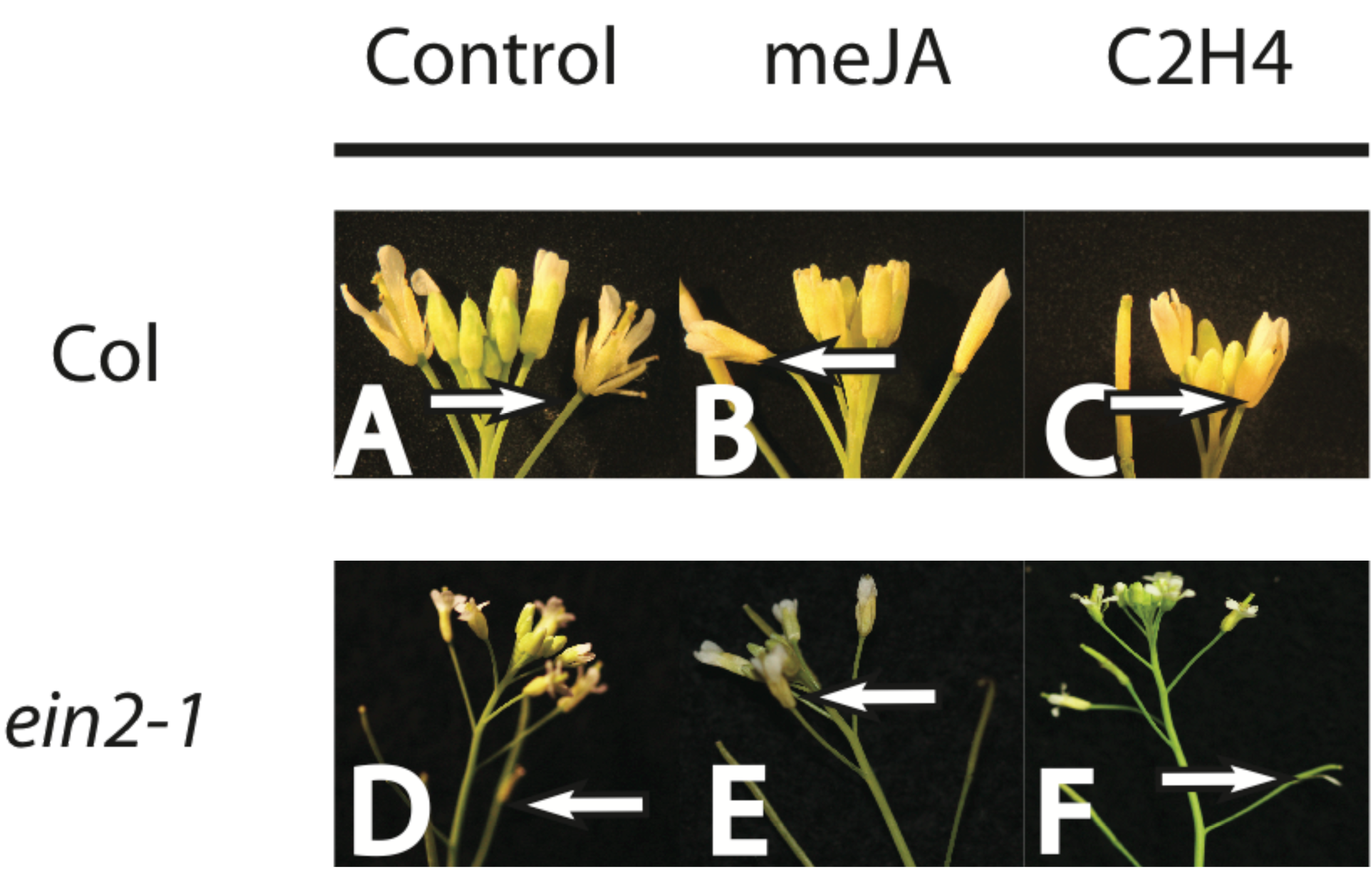

Supplement: Figure S4 — Leaf senescence of JA mutants and ethylene mutant ein2-1 .Comparison of leaves from wild type WS and Col to coi1-37, aos and ein2-1. Leaves were treated with 200 µM of meJA and 1 ppm ethylene as designated in Experimental Procedures. While aos displayed senescent tissues in response to both application of meJA and ethylene, coi1-37 and ein2-1 were only responsive to ethylene and meJA respectively. Wild type WS and Col displayed senescence with application of both meJA and ethylene. (TIF) [file pone.0060505.s004.tif]

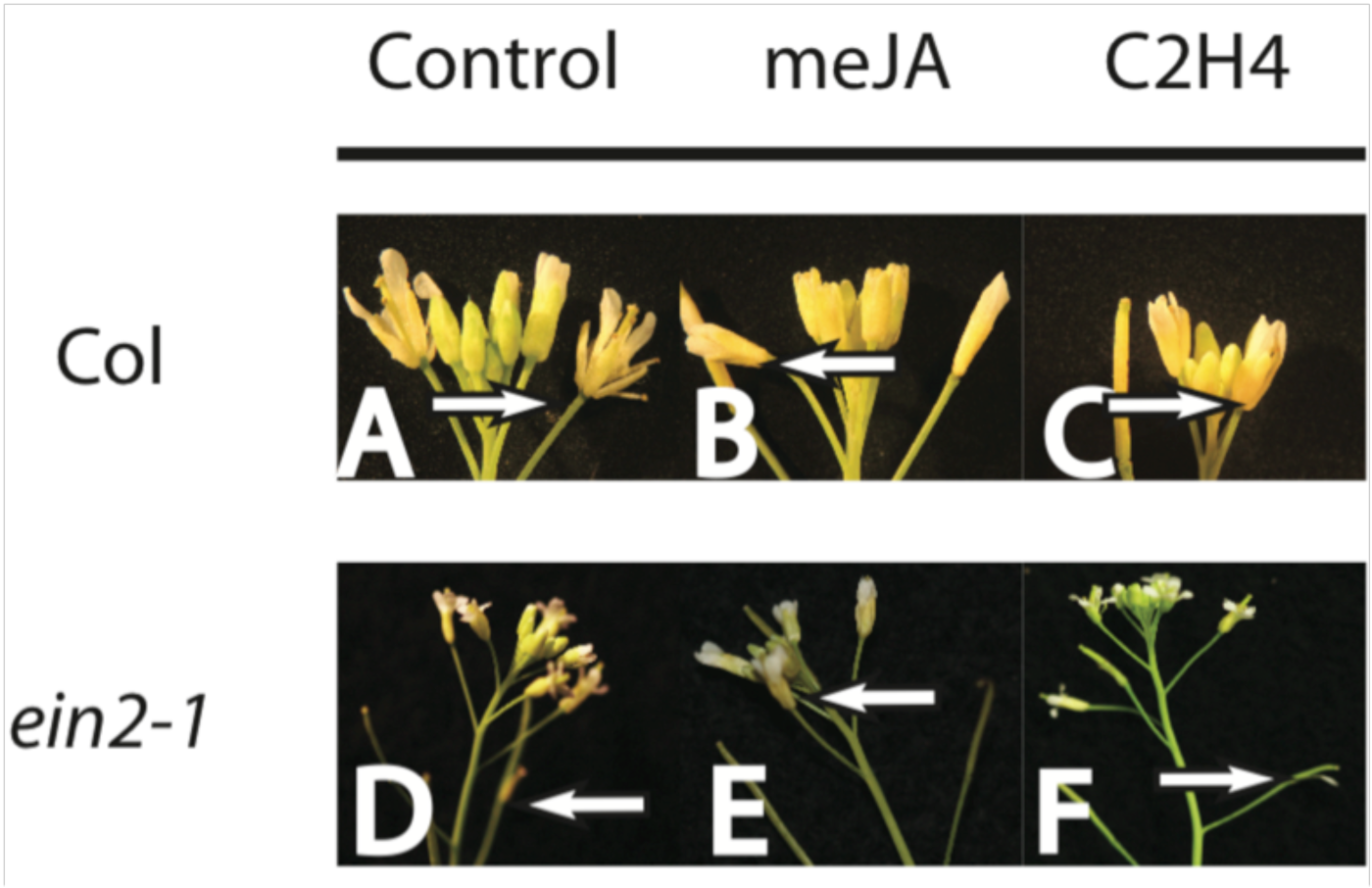

Supplement: Figure S5 — JA-dependent floral organ abscission. Comparison of floral organ abscission in Col (A-C) and ein2-1 (D-F). Whole plants are applied with 200 µM of meJA and 1 ppm of ethylene as described in Materials and Methods. Images are taken from primary inflorescence from Col and ein2-1. While Col was responsive to the applications of both meJA and ethylene (note the decreased numbers of flowers with petals still remain attached as well as total flowers), ein2-1 was only responsive to meJA (E). Both treatments accelerated floral organ abscission by position by 2-3 in wild type (B and C). The comparable degree of acceleration of abscission was observed in ein2-1 with responses to meJA (by flower position 3). Arrows indicate flower positions 4 (E) and 7 (D and F) of ein2-1 inflorescence respectively. (TIF) [file pone.0060505.s005.tif]
