# Supplementary material for: New Clothes for the Jasmonic Acid Receptor COI1: Delayed Abscission, Meristem Arrest and Apical Dominance
Source: PLoS One. 2013 Apr 1;8(4):e60505. doi: 10.1371/journal.pone.0060505 (PMC3613422; doi:10.1371/journal.pone.0060505)
Supplement: Table S1 — Segregation of delayed floral organ abscission trait in F2 population of coi1-37. (DOCX) [file pone.0060505.s006.docx]

**Table S1.**

**Segregation of delayed floral organ abscission trait in F2 population of *dab4-1/coi1-37*.**

| Maternal parent | Paternal parent | F1 | F2 segregation  (Wild type: delayed abscission) | X^2^ |
| --- | --- | --- | --- | --- |
| *dab4-1/coi1-37* -/- | WS | all wild type | 133:49 | 0.359 |
