# Supplementary material for: New Clothes for the Jasmonic Acid Receptor COI1: Delayed Abscission, Meristem Arrest and Apical Dominance
Source: PLoS One. 2013 Apr 1;8(4):e60505. doi: 10.1371/journal.pone.0060505 (PMC3613422; doi:10.1371/journal.pone.0060505)
Supplement: Table S2 — Segregation of delayed floral organ abscission trait and apical dominance traits in F1 population of test crosses. (DOCX) [file pone.0060505.s007.docx]

**Table S2. Segregation of delayed floral organ abscission trait and apical dominance traits in F1 population of test crosses.**

| Maternal parent | Paternal parent | Normal abscission: delayed abscission | Non-apical dominance : Apical dominance |
| --- | --- | --- | --- |
| *dab4-1/coi1-37* -/- | *dab4-1/coi1-37* +/- | 54 : 61 | 63 : 52 |

**Note: Total number of F1 plants examined for each phenotype is 115.**
