# Supplementary material for: New Clothes for the Jasmonic Acid Receptor COI1: Delayed Abscission, Meristem Arrest and Apical Dominance
Source: PLoS One. 2013 Apr 1;8(4):e60505. doi: 10.1371/journal.pone.0060505 (PMC3613422; doi:10.1371/journal.pone.0060505)
Supplement: Table S3 — Primers used for semi-quantitative and quantitative RT-PCR analysis in coi1-37. (DOCX) [file pone.0060505.s008.docx]

**Table S3. Primers used for RT-PCR analysis in *coi1-37***

| **Name of gene** | **Forward primer (5’to 3’)** | **Reverse Primer (5’to 3’)** |
| --- | --- | --- |
| *COI1* (At2g39940) | CTGAAGACCATTGTACGCTTATCC | CATCTCACGTTTGGACTGTACTGT |
| *AGL15* (At5g13790) | TAAATGCAGCCTCCAGAACACC | TTTCGCTGCTCGTGTTTGTTGT |
| *SHP1* (At3g58780) | GCAACACAATAACATGTACCTGCG | ATACACCGGATTCGTAAACTGTCG |
| *WUS* (At2g17950) | ATGAGTAGCCATGTCTATGGATCTATG | GTCTTGTTCCTTCACCCAAAAA |
| *MAF5* (At5g65080) | GTTGCTGAAAGAAAAGAACAAGGTTC | TTACTTGAGAAGCGGGAGAGTCT |
| *NAC2* (At5g39610) | GTTCTCTGTTTTACTCGGATCCTCTG | CCAAACGCAATCCAATTCTTCTG |
| *NAP* (At1g69490) | ATTACATGGGACCCGTCTCTCA | ACATCGCTTGACGATGATGGTT |
| *DDE1* (At2g06050) | GGGAGGTTTCATCTTTTGTCAA | TTTCGGGTACTTCACGTGGGAA |
| *AOS* (At5g42650) | GCGAGGTTGTTTGTGATTGAGA | CTAGCTTTCCTTAACGACGAGA |
| *DAD* (At2g44810) | AAATCGGTAAGGAGCTTCGGC | TATGGAGAACTCTCCGAGCTGTTT |
| *UBQ10* (At4g05320) | TGGTGGTTTCTAAATCTCGTCTCTGT | TCTATTACTAGAGGCCAACAATTGGACT |
| *ACT2* (At3g18780) | GTATCGCTGACCGTATGAGCAAA | TTTTCTGTGAACGATTCCTGGAC |
