# Supplementary figures and images for: Correction: New Clothes for the Jasmonic Acid Receptor COI1: Delayed Abscission, Meristem Arrest and Apical Dominance
Source: PLoS One. 2015 Mar 25;10(3):e0119063. doi: 10.1371/journal.pone.0119063 (PMC4373921; doi:10.1371/journal.pone.0119063)

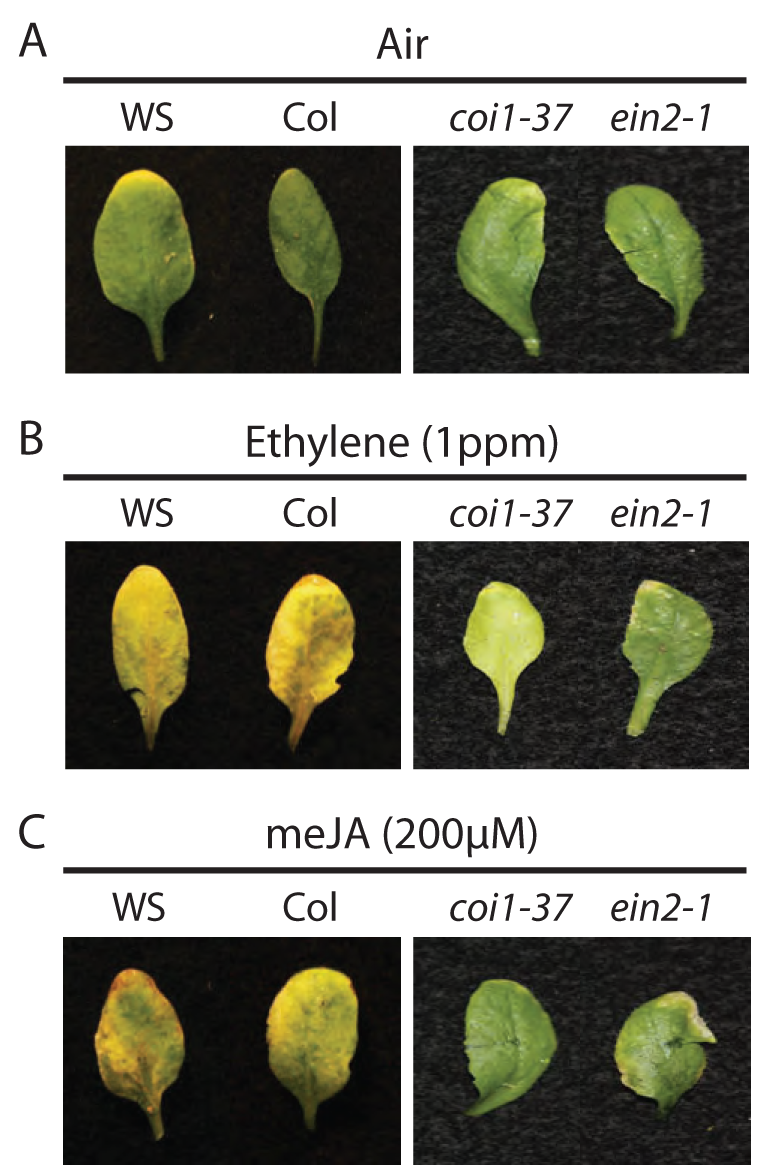

Supplement: S4 Fig — Comparison of leaves from wild type WS and Col to coi1–37, aos and ein2–1. Leaves were treated with 200 μM of meJA and 1 ppm ethylene as designated in Experimental Procedures. While aosdisplayed senescent tissues in response to both application of meJA and ethylene, coi1–37 and ein2–1 were only responsive to ethylene and meJA respectively. Wild type WS and Col displayed senescence with application of both meJA and ethylene. (TIF) [file pone.0119063.s001.tif]
